# Modeling the importance of life exposure factors on memory performance in diverse older adults: A machine learning approach

**DOI:** 10.1002/alz.70428

**Published:** 2025-08-20

**Authors:** Evan Fletcher, Marianne Chanti‐Ketterl, Emily Hokett, Yi Lor, Umesh Venkatesan, Ruijia Chen, Omonigho M. Bubu, Rachel Whitmer, Paola Gilsanz, Zvinka Z. Zlatar

**Affiliations:** ^1^ Department of Neurology School of Medicine University of California Davis Davis California USA; ^2^ Department of Psychiatry and Behavioral Sciences School of Medicine Duke University Durham North Carolina USA; ^3^ Department of Neurology Columbia University New York New York USA; ^4^ Department of Public Health Sciences University of California Davis Davis California USA; ^5^ Moss Rehabilitation Research Institute & Department of Rehabilitation Medicine Sidney Kimmel Medical College at Thomas Jefferson University Philadelphia Pennsylvania USA; ^6^ Department of Epidemiology Boston University School of Public Health Boston Massachusetts USA; ^7^ Departments of Psychiatry Neurology and Population Health and NYU Neuroscience Institute NYU Grossman School of Medicine New York New York USA; ^8^ Department of Public Health Sciences and Department of Neurology University of California Davis Davis California USA; ^9^ Kaiser Permanente Division of Research Oakland California USA; ^10^ Department of Psychiatry University of California San Diego La Jolla California USA

**Keywords:** episodic memory, life exposure factors, regression tree models, SHAP values, XGBoost

## Abstract

**INTRODUCTION:**

Many health life exposure factors (LEFs) influence cognitive decline and dementia incidence, but their relative importance to episodic memory (an early indicator of cognitive decline) among diverse older adults is unclear. We used machine learning to rank LEFs for memory performance in a large and diverse US cohort.

**METHODS:**

Kaiser Healthy Aging and Diverse Life Experiences (KHANDLE) and Study of Healthy Aging in African Americans (STAR), participants underwent neuropsychological testing and answered questionnaires about multiple LEFs. XGBoost and Shapley Additive exPlanation values ranked the importance of factors influencing cross‐sectional episodic memory in the full sample and by sex and ethnic group.

**RESULTS:**

Among 2245 adults (mean age: 74 years; range 54–90), age, sex, education, volunteering, income, vision, hearing, sleep, and exercise contributed to memory performance regardless of group stratification.

**DISCUSSION:**

This innovative methodology can help identify risk factors important for memory performance and guide future dementia risk reduction interventions among older adults.

**Highlights:**

- This work uses a regression tree machine learning model (XGBoost) with highly interpretable Shapley Additive exPlanation values to analyze impacts of 12 life exposure factors plus age, sex and ethnoracial identity on episodic memory outcome.
- This approach has valuable properties, including the ability to implicitly account for variable interactions, non‐linear relations with outcome, and missing values.
- Age, sex, education, income, volunteering, exercise, hearing and vision, and sleep (quality and duration) have important impacts on memory outcome in a combined model and in stratified models regardless of ethnoracial identity.
- We also demonstrate individualized models for subgroups of participants, showing how life exposure factors vary in importance between divergent populations and suggesting an approach to personalized interventions.
- This approach can be valuable for both policy decisions and individualized interventions to support healthy cognitive aging.

## 1 BACKGROUND

The 2024 Lancet Commission report on dementia prevention indicated that up to 45% of Alzheimer's disease and related dementias (ADRD) cases worldwide could be delayed or prevented by addressing 14 potentially modifiable health life exposure factors.^1^ These included education attainment, high low‐density lipoprotein cholesterol, depression, traumatic brain injury (TBI), physical inactivity, diabetes, smoking, hypertension, obesity, excessive alcohol use, social isolation, air pollution, and vision/hearing impairment. Many of these dementia risk factors (hereafter life exposure factors [LEFs]) are shaped by sociocultural background, life experiences, and access to resources, highlighting the need to understand their varied influences on cognitive health across different ethnic/racial (hereafter ethnic) groups. For example, ADRD prevalence is significantly lower among Asian and White older adults compared to Black/African American and Hispanic/Latino(as)x (hereafter Black, Hispanic) populations.^2^, ^3^ Improving these LEFs may enhance cognitive resilience and lessen ADRD risk.^4^ Additionally, the proportion of ADRD cases attributable to LEFs is nearly 6% higher in men than in women and varies by ethnicity in the United States.^2^ Therefore, recognizing the varying importance of ADRD risk factors across different sex and ethnic contexts is essential.^2^, ^5^, ^6^, ^7^, ^8^

It is equally important to examine LEFs related to cognitive performance, rather than solely focusing on ADRD diagnosis, as this approach offers a more comprehensive understanding of the cognitive aging processes and potential disparities in ADRD risk profiles across different groups. Different environments and cultural practices can influence lifestyle habits, such as smoking and alcohol consumption, sleep and exercise routines, and attitudes toward community engagement and health‐care–seeking behaviors, which can vary across ethnic groups.^2^, ^9^ Additionally, cardiovascular risk factors and TBI, which significantly influence brain health and ADRD risk,^2^, ^5^, ^6^, ^7^, ^8^ are more prevalent and have been consistently linked to lower cognitive performance, particularly among older Black^7^, ^10^ and Hispanic adults,^11^, ^12^ compared to White adults. Furthermore, sex influences how these factors affect cognitive function;^10^, ^13^, ^14^ for example, cardiovascular conditions are more strongly predictive of cognitive decline in women than in men.^13^, ^14^ These findings suggest tailored socioenvironmental and behavioral interventions may help groups at high risk of ADRD maintain cognitive health into late life and decrease ADRD risk.

Common methods for quantifying the relative importance of risk factors may have limited capacity to model complex interactions among them. For example, population attributable fractions (PAFs)^1^ calculate the percentage reduction in new dementia cases if a specific factor were eliminated.^15^ However, these calculations do not fully account for factor interactions. More generally, because such interactions are difficult to model explicitly, multifactorial techniques may be more appropriate for many outcomes of interest.^16^, ^17^ Consequently, while ordinary least squares regression is widely used and easily interpretable, it does not fully account for all factor interactions, covariances, and non‐linear relations between variables and outcomes.^18^

To address these issues, we used an interpretable framework incorporating a machine learning regression‐tree approach (XGBoost)^19^ with Shapley Additive exPlanation (SHAP) values.^20^, ^21^ XGBoost accounts for variable interactions, non‐linear relations, and missing predictor data.^19^ SHAP values provide both model‐wide and individualized importance rankings of predictor variables.^20^ This framework has been used to model outcomes based on large numbers of predictors (e.g., Yi et al.^22^ and Guimbaud et al.^23^), but to our knowledge, it has not been applied to cognitive performance among diverse older participant samples. We therefore investigated the contribution of 12 LEFs, identified in recent studies and available in our study database,^1^, ^2^ along with age, sex, and self‐reported ethnicity, on memory performance in a large, diverse US cohort. XGBoost quantified each variable's contribution to the memory outcome, assessing both global importance rankings across the entire sample and local importance values specific to each participant, stratified by both ethnicity and sex. We expected to see differences in risk factor rankings across groups because the literature suggests that there are sex and ethnic differences in the prevalence and importance of these factors for both memory and dementia risk.^6^, ^24^, ^25^, ^26^

## 2 METHODS

### 2.1 Study population

In this study, we used data from the Kaiser Healthy Aging and Diverse Life Experiences (KHANDLE)^27^ and the Study of Healthy Aging in African Americans (STAR),^28^ harmonized cohorts of long‐term members of Kaiser Permanente Northern California, an integrated health‐care delivery system. Both studies administered the same neuropsychological assessments and questionnaires. Briefly, KHANDLE includes community‐dwelling adults in the San Francisco Bay and Sacramento regions of California who were age ≥ 65 as of January 1, 2017. Exclusion criteria include an electronic medical record diagnosis of dementia or a diagnosis of poor health that could interfere with study participation. The KHANDLE study participants were selected to achieve a balance of self‐identified ethnic identities among Asians, Blacks, Hispanics, and Whites. The STAR study includes community‐dwelling Black adults from the San Francisco Bay Area who were at least 50 years of age on January 1, 2018. The same exclusion criteria applied as in the KHANDLE study. More information about the study design of both studies has been published elsewhere.^27^, ^29^, ^30^ Our study used cross‐sectional data collected at wave 1 of each cohort. After 23 exclusions of missing data for the episodic memory outcome and two for unknown ethnic identity, the analytical sample included 2245 participants, comprising 840 males and 1405 females, with 413 Asian, 987 Black, 349 Hispanic, and 496 White adults.

**RESEARCH IN CONTEXT**

1. **Systematic review**: We reviewed the literature from databases (e.g., PubMed, Google Scholar) to identify studies on modifiable risk factors for dementia. Existing studies used traditional epidemiological methods such as population attributable risk and linear regressions. This article extends the literature by using XGBoost decision trees to compute the relative importance of life exposure factors, accounting for age, education, and sex differences, on episodic memory in an ethnoracially diverse sample.
2. **Interpretation**: In 2245 adults (413 Asian, 987 Black, 349 Hispanic, and 496 White), education, income, volunteering, exercise, hearing, vision, and sleep significantly contributed to memory performance across all ethnoracial and sex groups. This suggests that structural factors like higher education, leading to higher income; more volunteering opportunities; and better sleep, are crucial for memory across diverse cohorts. This machine learning approach may inform personalized medicine interventions to reduce Alzheimer's disease and related dementias risk and sustain healthy cognitive aging.
3. **Future directions**: Further research with a larger national sample and more comprehensive dementia risk factors is needed to determine whether machine learning can accurately predict individual cognitive decline risk.

### 2.2 Outcome: episodic memory

Episodic memory (hereafter memory) was equally assessed across studies using the Spanish and English Neuropsychological Assessment Scales (SENAS), administered in English or Spanish during wave 1. SENAS is a series of cognitive tests developed using item response theory (IRT) methodology to ensure valid comparisons of cognitive aging across diverse ethnic and linguistic groups.^31^, ^32^, ^33^ Verbal memory composite scores (in *z* score metric based on the group mean) were derived from the SENAS memory domain, comprising a standard word list learning test with five learning trials and a short‐delay free recall trial after distraction. The five learning trials and the delayed recall trials are ordinal items modeled with a graded‐response IRT model. For complete details, see Mungas et al.^32^ and supporting information (https://doi.org/10.1037/1040‐3590.16.4.347.supp/). Memory is analyzed as a continuous measure of memory performance rather than a binary for incidence of ADRD. This allows us to examine more nuanced relationships among our variables. Importantly, the Livingston and Nianogo papers^1^, ^2^ assess ADRD risk based on diagnosis, which is subject to variability across clinicians and settings. Using a continuous cognitive outcome mitigates that subjectivity, strengthening the model. Our approach, leveraging XGBoost and SHAP values, quantifies each variable's marginal contribution while considering all other variables, including interactions and non‐linear relations. Differences in variable importance rankings by this method could therefore occur when comparing to rankings by regression methods modeling categorical outcomes.

### 2.3 Life exposure factors related to memory

LEFs selected for this study were based on previous research^1^, ^2^ and data availability at the baseline cognitive assessment of the KHANDLE and STAR cohorts. Consistent with earlier approaches, we included age as a risk factor because it is a powerful predictor of cognition and allows us to examine its interactions with other variables. To conduct both stratified and full analyses using a single trained model, we coded a self‐selected sex variable (1 for males, 2 for females) and created one‐hot encoded variables (1 = yes, 0 = no) for self‐identified ethnic categories, including Asian, Black, Hispanic, and White. All LEFs assessed in this study were self‐reported and collected from harmonized questionnaires in both KHANDLE and STAR studies at wave 1. Where possible, our variables consisted of a range of graded responses, whether in terms of frequency or quality, avoiding cutoffs that could introduce arbitrary judgments. However, an exception was made for alcohol use, for which we used an algorithm to compute weekly consumption relative to the Centers for Disease Control (CDC) standard. Smoking, volunteering, and TBI were available only as binary indicators.

To capture amounts of light and vigorous exercise, participants were asked to rate how frequently they currently participated in the following activities: (1) light exercise or sports (walking, dancing, softball, bowling, etc.) and (2) vigorous exercise or sports (cycling, jogging, swimming laps, tennis, etc.). The answer choices for each of these two questions were: 1 = every day, or almost every day || 2 = several times a week || 3 = several times a month || 4 = several times a year || 5 = never. Physical activity variables in our model were reverse coded, ranging from 5 = every day, or almost every day, to 1 = never. As such, higher scores represented more frequent engagement in either light or vigorous exercise. Although the “vigorous” and “light” exercise questions contain activities that are mutually exclusive, they may or may not overlap in terms of physical intensity. We are considering both variables independently to assess the separate contribution of each to memory, given literature suggesting differential associations between light and vigorous activity levels with cognition and brain health.^34^, ^35^, ^36^, ^37^, ^38^, ^39^

For hearing, we used a 5‐point scale of self‐reported hearing quality using hearing aids if needed: “excellent” (5), “very good” (4), “good” (3), “fair” (2), and “poor” (1).

For vision, a 6‐point scale was used for self‐reported ratings for vision quality with corrective lenses if needed: “excellent” (6), “very good” (5), “good” (4), “fair” (3), and “poor” (2), and “legally blind” (1).

We defined excessive alcohol use with the CDC threshold of 7 drinks per week for women and 14 drinks weekly for men (https://www.cdc.gov/drinklessbeyourbest/drinkingless.html). We chose to apply the CDC standard for excessive alcohol consumption as it provides a widely accepted, evidence‐based definition that ensures consistency in identifying excessive alcohol use across studies and populations.

To capture tobacco use, participants were asked at baseline if they had ever smoked cigarettes, with a binary response (yes/no).

For sleep, participants were asked to self‐report the average number of hours they slept per night over the past month. Sleep quality was measured with a numerical sum score based on a modified version of the Pittsburgh Sleep Quality Index (PSQI).^40^ The index includes a summation of six sleep variables (e.g., daytime sleepiness, bad dreams, pain), with a lower score indicating better sleep quality.

Participants were asked if they had volunteered (yes or no) for religious, educational, health‐related, or other charitable organizations in the past 12 months at baseline.

Education was operationalized as years of schooling from 0 to 20.

Annual income was captured using 13 categories of annual income range, with $0 to $9999 being the lowest category and > $125,000 the highest.

To account for TBIs, participants were asked if they ever had a head injury that required medical care with a binary response (yes/no).

### 2.4 Statistical analysis

Analyses were performed in RStudio version 2024.12.0+467. These included the R packages tidyverse_2.0.0, tidymodels_1.0.0, SHAPforxgboost_0.1.3, shapviz_0.9.2, and xgboost_1.7.3.1. To understand sex and ethnic differences of variables in our sample, we performed analysis of variance (ANOVA) and pairwise Tukey analyses for the 12 LEF risk factors (light exercise, vigorous exercise, hearing, vision, alcohol use, tobacco use, sleep duration, sleep quality, volunteering, education, income, and TBI). We then used the XGBoost Regression Tree algorithm, a type of eXtreme Gradient Boosting machine learning, to assess the influence of the 12 risk factors listed above plus age, sex, and ethnicity on memory outcomes. All the multi‐level variables (i.e., other than binary indicators) were converted into *z* scores with a mean of 0 and a standard deviation (SD) of 1 prior to analyses using the R scale() function from the base package (4.2.2). We also explored the influence of these 12 factors in separate analyses stratified by sex and ethnicity (Asian, Black, Hispanic, and non‐Hispanic White). Here, our final XGBoost model, trained on the full set, was used to make predictions within stratified subsets obtained by selecting a single value of the relevant indicator variable. The gradient boosted regression tree model is particularly useful as it effectively handles non‐linear relationships, interaction effects, and missing predictor values.^19^

#### 2.4.1 XGBoost tree ensemble model training and performance validation

Our model was trained using a 75%/25% split of the data into training and test sets. We performed this for four randomly chosen splits, then choosing the model having the smallest difference of *R* ^2^ fit for training versus test sets, suggesting the least overfitting in the training set and best generalizability to the test set. Training consisted of tuning seven model hyperparameters, notably including number of trees, maximal tree depth, and number of variables to sample at each tree split. These are aimed at minimizing overfitting while allowing the model to adequately learn the relationships in the data. A complete list of hyperparameters is given in the supporting information. Hyperparameter tuning involved a 5‐fold cross‐validation in the training data. In each fold, 80% of the training set was used to find the best combination of hyperparameter values from a representative sampling grid of possible values, while the remaining 20% tested the model's performance with these hyperparameters. The best overall set of hyperparameters was chosen for the model fit, which was then tested on the 25% test portion of the data to verify accuracy. The best fit was obtained by minimizing the root mean squared error (RMSE) of model predictions in the training set. The fit quality was measured using the coefficient of determination (*R* ^2^) for comparison to regression. We then performed parallel multiple least squared regression on the same training and test sets for use as a standard comparison to our XGBoost model fits.

#### 2.4.2 SHAP value interpretation

To interpret the model, we used SHAP values.^21^ SHAP values provide multiple levels of information, including the global importance for the entire sample of model variables and the local, or contextual, impacts, depending on the particular values for each participant.^23^ In this paper, therefore, “local” and “context” refer interchangeably to the individual array of variable values particular to each participant, while “global” refers to the effects of variables in the entire model. At the global level, “importance” describes the average absolute marginal contribution of a variable to memory, considering all possible contexts of the other feature values, including when features are missing.^20^ At the local level, “impact” applies to the signed (positive or negative) marginal contribution of a variable to memory for a single participant.

SHAP values provide global explanations through feature importance rankings, analogous to the magnitudes of ß regression coefficients, indicating the magnitude of a variable's impact on the outcome across the entire dataset. They also provide local explanations, showing how each risk factor's signed impact varies for an individual participant, considering the other variable values of that participant. Notably, the model may predict different rankings of summed signed variable importance than the global ranking for the whole population. This can yield valuable individual insights. In sum, the analysis includes main effects and interactions with other features, modeling all possible feature interactions in a way that traditional regression models cannot.^20^

We used SHAP bee swarm plots to provide multiple layers of interpretability. These plots depict the overall importance of variables across the population, the importance of each variable (LEF plus age and sex) in the context of all the other variables for individual participants, and the distributions of SHAP values corresponding to raw values of each variable.^20^ Additionally, we used waterfall plots to illustrate the contributions of each variable to memory prediction for subgroups of participants. The waterfall plot structure highlights the positive and negative contributions that sum to predict memory for an individual subgroup.

## 3 RESULTS

### 3.1 Demographic characteristics

Table 1 presents the demographic characteristics of the participants by sex and ethnic groups. The study included 2245 adults aged ≥ 50, 37% males. The mean age of the sample was 74.1 (SD = 8.0) years with an average education of 14.6 years (SD = 3.0). Four participants did not identify with any ethnic group but were kept in the sex‐stratified sample. Ethnic categories included: 18% Asian, 44% Black, 16% Hispanic, and 22% White adults.

**TABLE 1:** Demographic characteristics.

Characteristic	Full cohort	Male	Female	Asian	Black	Hispanic	White
***N* (%)**	2245	840 (37.4%)	1405 (62.6%)	413 (18.4%)	987 (44.0%)	349 (15.5%)	496 (22.1%)
**Outcome**							
Memory	0.17 (0.88)	−0.14 (0.81)	0.36 (0.87)	0.25 (0.89)	0.19 (0.85)	−0.04 (0.87)	0.21 (0.92)
**Life exposures (predictors)**							
Age years, mean (SD)	74.0 (8.0)	74.7 (7.6)	73.6 (8.2)	75.6 (6.6)	71.2 (8.5)	76.0 (6.4)	76.9 (7.2)
Education years, mean (SD)	14.6 (3.0)	14.9 (3.1)	14.4 (3.0)	15.6 (2.6)	14.4 (2.6)	13.1 (4.0)	15.2 (2.9)
Income range category mean score, mean (SD)	8.7 (2.9)	9.6 (2.6)	8.2 (2.9)	9.6 (2.8)	8.5 (2.9)	7.9 (3.0)	9.0 (2.8)
Exceed alcohol threshold, % yes	9.7%	11.8%	8.6%	7.2%	5.1%	11.3%	22.2%
Smoke (ever), % yes	44.9%	51.7%	40.9%	32.2%	45.1%	48.9%	52.5%
Volunteer in past year, % yes	48.1%	42.9%	51.1%	45.3%	51.6%	37.8%	50.5%
Hearing (levels) 0 = poor to 4 = excellent	2.2 (1.1)	2.0 (1.1)	2.3 (1.1)	2.1 (1.0)	2.4 (1.1)	2.1 (1.1)	2.0 (1.1)
Vision, mean (SD) 0 = blind to 5 = excellent	3.2 (0.9)	3.2 (0.9)	3.2 (0.9)	3.1 (0.9)	3.1 (0.9)	3.1 (1.0)	3.3 (0.9)
Hours of sleep per night, mean (SD)	6.5 (1.4)	6.5 (1.4)	6.4 (1.4)	6.4 (1.3)	6.2 (1.4)	6.6 (1.3)	7.1 (1.3)
Sleep quality, mean (SD)	4.6 (3.3)	4.1 (3.2)	4.8 (3.3)	4.1 (3.2)	5.0 (3.4)	4.2 (3.2)	4.5 (3.1)
Light exercise (levels) 1 = never to 5 = everyday	2.8 (1.3)	2.8 (1.3)	2.8 (1.3)	3.0 (1.3)	2.6 (1.3)	2.7 (1.4)	2.9 (1.2)
Vigorous exercise, mean (SD) 1 = never to 5 = everyday	1.1 (1.4)	1.4 (1.5)	1.0 (1.3)	1.2 (1.4)	1.1 (1.4)	1.0 (1.4)	1.2 (1.4)
Traumatic brain injury, % yes	21.7%	26.8%	18.6%	17.7%	17.4%	29.5%	27.8%

Abbreviation: SD, standard deviation.

As mentioned, the XGBoost model assigns local SHAP values even to missing variables, seen as gray color in the plots. Among our variables, alcohol had the highest number of missing values (601 = 26.7%), followed by income (252 = 11.2%) and TBI (63 = 2.8%). A full table of missing values is provided in Table S1 in supporting information.

ANOVA and Tukey honestly significant difference (HSD) detailed results are provided in Table S2a–d in supporting information. ANOVA by sex differences (Table S2c) indicated that males were significantly older, had lower memory scores, higher education attainment, more frequent vigorous exercise, and higher income than females. Females had better hearing and better sleep quality. ANOVA by ethnicity (Table S2d), indicated that all variables but vigorous exercise were statistically significant. Table S2b shows Tukey HSD pairwise comparisons for ethnicity. Only the significant differences are shown. Non‐significant differences included memory scores between Asian and Black or White adults, and between Black and White adults, and sleep quality between Asian and Hispanic participants, Asian and White participants, and White and Hispanic participants.

### 3.2 XGBoost results

**FIGURE 1:**
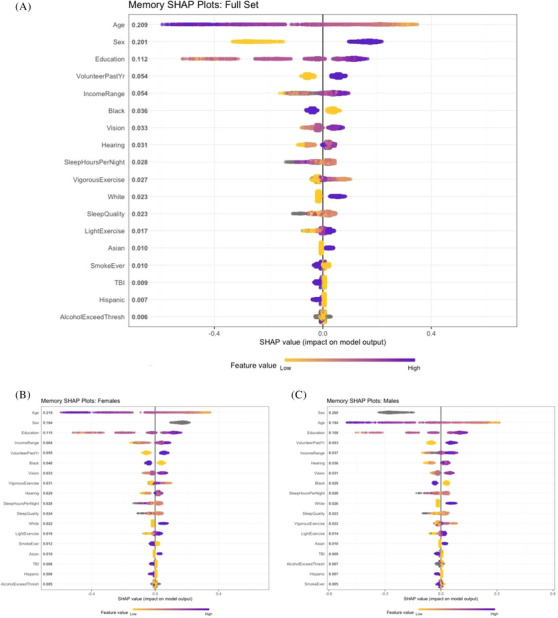
Shapley bee swarm plots for episodic memory outcome. Global importance (mean absolute SHAP value) is listed in descending order on the left. Horizontal plots to the right of the vertical line (x=0.0) indicate a positive influence on memory performance, and those left of the line suggest a negative influence on the outcome. Color codings of yellow to blue indicate ranges from low to high of raw values for each variable. For binary sex, yellow (low) = male and blue (high) = female. For ethnicity indicator variables, yellow = not in this subgroup, and blue = in this subgroup. For other binary variables, yellow = no, blue = yes (e.g., SmokeEver). Missing values are coded gray. A, Plots for full model. B, Plots for females. C, Plot for males. SHAP, Shapley Additive exPlanation; TBI, traumatic brain injury.

The best fit of the full XGBoost model (Figure 1) to training data had metrics of RMSE = 0.694, and *R* ^2^ = 0.371. Corresponding metrics for test data were RMSE = 0.735 and *R* ^2^ = 0.326. For comparison, multivariate regression fits had *R* ^2 ^= 0.257 on the same training set and *R* ^2 ^= 0.311 on the test set. Although XGBoost outperformed regression on the training set, as expected, it also did slightly better on the test set consisting of data not seen in training. This supports XGBoost's model robustness. Optimal hyperparameter values for the XGBoost model are included in Table S3 in supporting information.

Figure 1A displays the bee swarm plots for memory for the entire sample. Global importance (non‐negative contributions to the overall model) is displayed vertically on the *y* axis, and local impacts of each variable (positive or negative values for each participant) are displayed horizontally. The color gradient in the plot represents the raw feature values, with yellow denoting lower values and blue–purple representing higher values. Local impacts (horizontal position to the right or left of *x* = 0) record the different local impacts of a variable for individual participants. Age exhibited the highest global importance, with a SHAP value of 0.209. Higher age (purple–blue in the plots) had negative local impacts on memory, while younger age had positive impacts. Sex followed closely, with a global importance value of 0.201, and local impacts (negative for male = yellow, positive for female = blue) suggesting strong sex‐based differences. Education, volunteering, and income also demonstrated relatively high global impacts with SHAP values of 0.112, 0.054, and 0.054, respectively. Ethnic variables, such as Black, White, Asian, and Hispanic, exhibited varying degrees of global ranking, with global importance values ranging from 0.036 to 0.007. For example, being Black (“yes” = blue color) negatively impacted memory performance, while not being Black (yellow) had a positive impact. Similarly, sensory health factors like vision and hearing contributed modestly (0.033 and 0.031 global importance, respectively). Sleep hours per night (0.028), vigorous exercise (0.027), and sleep quality (0.023) also had moderate global importance. Notably, lifestyle and health‐related risk factors such as smoking history (0.010), TBI (0.009), and alcohol consumption exceeding the CDC threshold (0.006) had smaller global importance. This visualization confirms the dominant influence of demographic factors like age and sex while also capturing the contributions of socioeconomic, health, and lifestyle characteristics to memory‐related predictions.

Some nuance in these variables is observed. For instance, poor hearing (yellow) has an unambiguous negative local impact, but excellent hearing (blue) shows less positive impact than slightly worse values (i.e., “very good” or “good” rather than “excellent”; light to dark purple). Sleep hours per night also exhibit nuance, with the highest sleep hours (blue) having negative local impacts on memory, while midrange values (orange) have positive local impact. Similar patterns appear in vigorous exercise, with moderate frequency participation making a more positive impact on memory performance than either never exercising (yellow) or exercising almost daily (purple). Such nuanced patterns may suggest non‐linear relations of variables to their impact on memory. The global importance for light exercise is 0.017 and for vigorous exercise 0.027. Furthermore, light exercise shows more linear‐appearing local impacts, with the most positive impacts for the highest frequency (blue) and negative impacts for the lowest, whereas vigorous exercise shows an inverted U response with memory performance, highlighting the value of looking at these two variables separately. Although some risk factors have missing raw values for certain participants (represented in gray), these still have SHAP values, because XGBoost learns optimal decisions in the absence of variables, as previously explained.

Figure 1B,C present bee swarm plots for models stratified by sex. In each figure, the sex variable has only the value 1 and is plotted in gray. The relative importance is still valid, however. Age is the strongest global importance factor in both groups, followed by education, volunteering, and income, highlighting the role of socioeconomic and social engagement factors. Sensory health, sleep, exercise, and ethnic variables also contribute moderately. Key differences emerge in the impact of specific factors. Age has a higher relative global ranking in females (0.219; Figure 1B) compared to males (0.194; Figure 1C). Education also plays a larger role for females (0.115 vs. 0.108). Income has higher global importance in females (0.064) compared to males (0.037). Sensory health appears to flip in importance. In males, hearing (0.036) is more important than vision (0.031), while in females, vision (0.033) has higher importance than hearing (0.029). The Black indicator variable has higher global importance among females (0.040) than males (0.029). Vigorous exercise has higher relative importance among females (0.031) than males (0.022).

**FIGURE 2:**
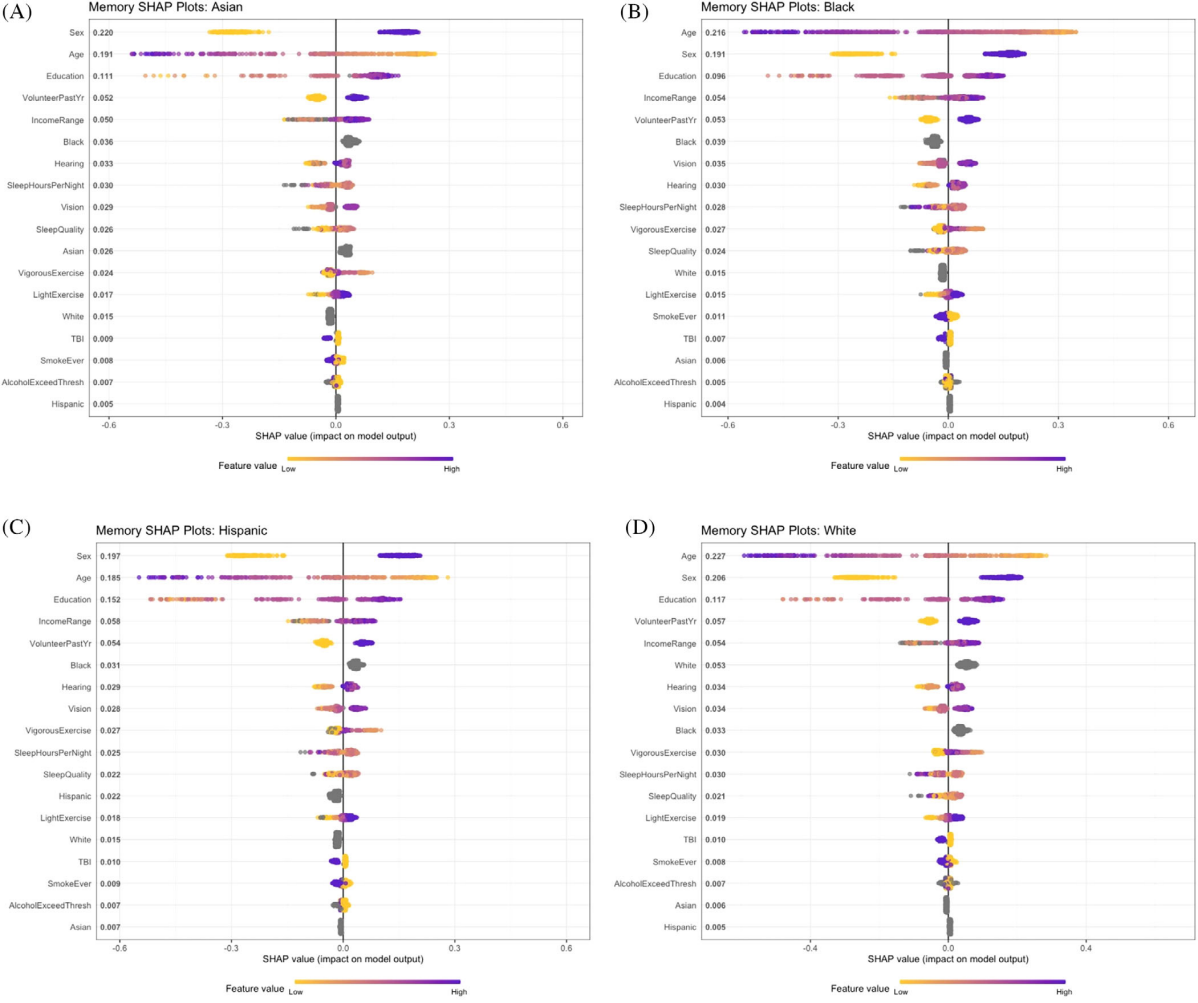
Shapley plots for memory, by ethnicity. Note: Each figure depicts the global *importance* (mean absolute SHAP value) listed in descending order on the left (*y* axis). Values to the right (*x* axis) of the vertical line (x=0.0) indicate a positive impact, or influence, on memory performance, and those to the left of the line suggest a negative influence on the outcome. Color coding of yellow to purple indicates ranges from low (yellow) to high (purple) of raw values for each variable. For binary variables such as sex, yellow (low) = male, and blue (high) = female. For other binary variables, yellow = no, blue = yes (e.g., ever smoked yes or no, Asian ethnicity yes or no). Missing values are coded gray. A, Asian; (B) Black; (C) Hispanic; (D) White. AlchoholExceedThresh, Alcohol intake exceeding the threshold established by the Centers for Disease Control. SHAP, Shapley Additive explanation; TBI, traumatic brain injury.

Figure 2 displays bee swarm plots stratified by ethnic category. Age and sex are the two variables with the most global importance in all categories, with age being most important among Black and White participants, and sex the most important in the other two groups. After these, education, volunteering, and income follow in all groups, with education being the most important and volunteering having about the same importance as income. Hearing and vision have mid‐rank importance in all plots, with sleep hours, sleep quality, and vigorous exercise slightly below them.

**FIGURE 3:**
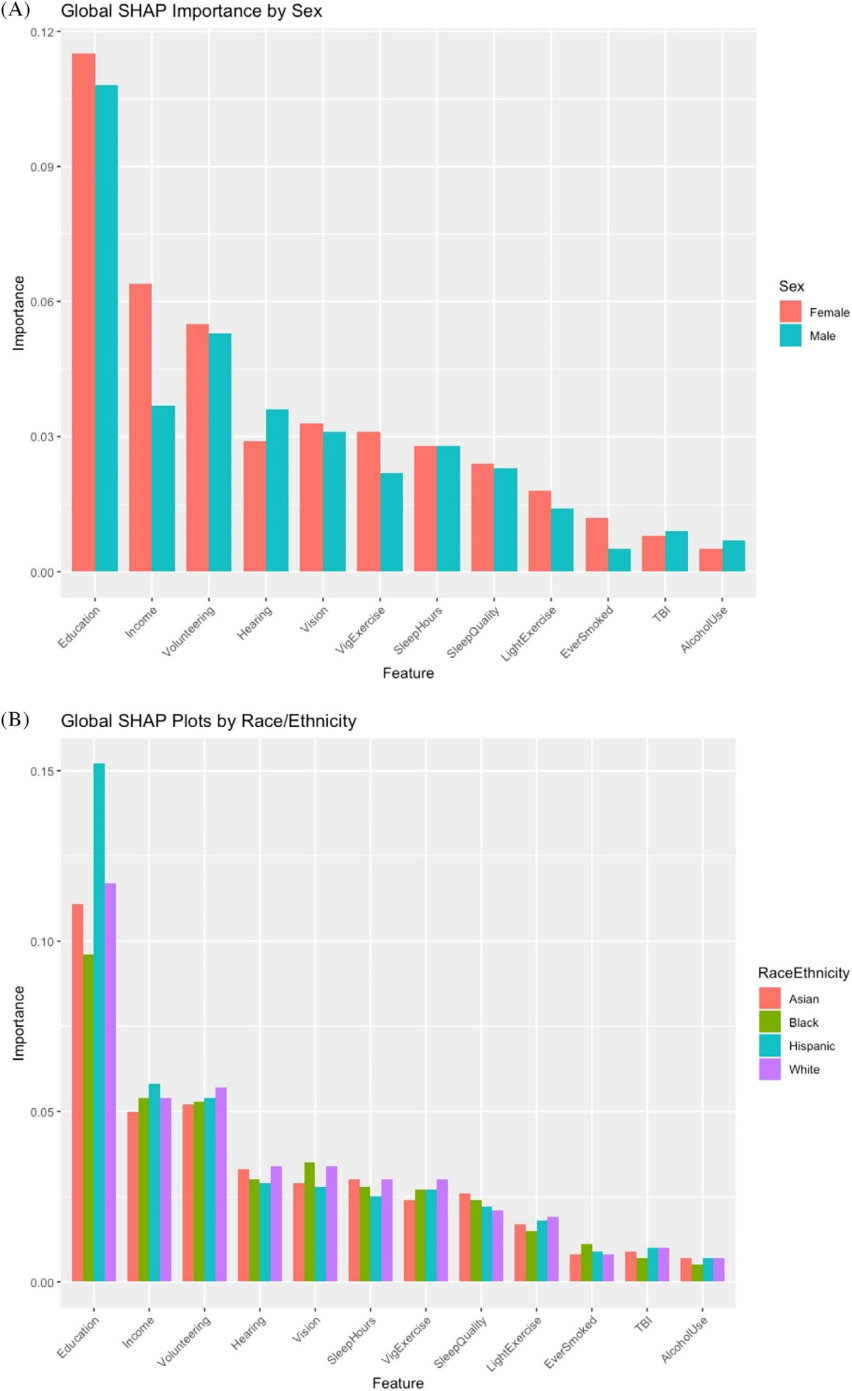
Comparisons of SHAP global importance (mean absolute SHAP values) of lifestyle exposure factors to memory performance stratified by sex (A) and ethnicity (B). SHAP, Shapley Additive exPlanation; TBI, traumatic brain injury.

Figure 3 compares the global importance of LEFs by sex and ethnic group, reorganized to highlight specific risk factor importance across sex (Figure 3A) or ethnic categories (Figure 3B). It depicts comparative rankings of all variables in each category (e.g., male or female) and within each variable, its relative importance across sex or racial/ethnic categories. We do not show age, sex, or ethnic indicators. The figures display relatively uniform global importance of any given variable across sex or ethnicity, with notable exceptions of education being 1.5 times more important for Hispanics than other ethnicities and income being 1.7 times more important for females than males. They also reveal an importance ranking of all the variables that remains roughly constant across sex or ethnicity. They suggest tiers of importance that approximately hold for both sexes and all ethnicities. Education is the most important factor, with a slightly greater impact on females and Hispanic participants. Income is the second strongest predictor, followed by volunteering, which shows higher importance in females and varies slightly across ethnic groups. Hearing, vision, sleep hours, vigorous exercise, sleep quality, and possibly light exercise are of moderate global importance by both sex and ethnicity. Smoking, TBI, and alcohol use have uniformly low importance.

**FIGURE 4:**
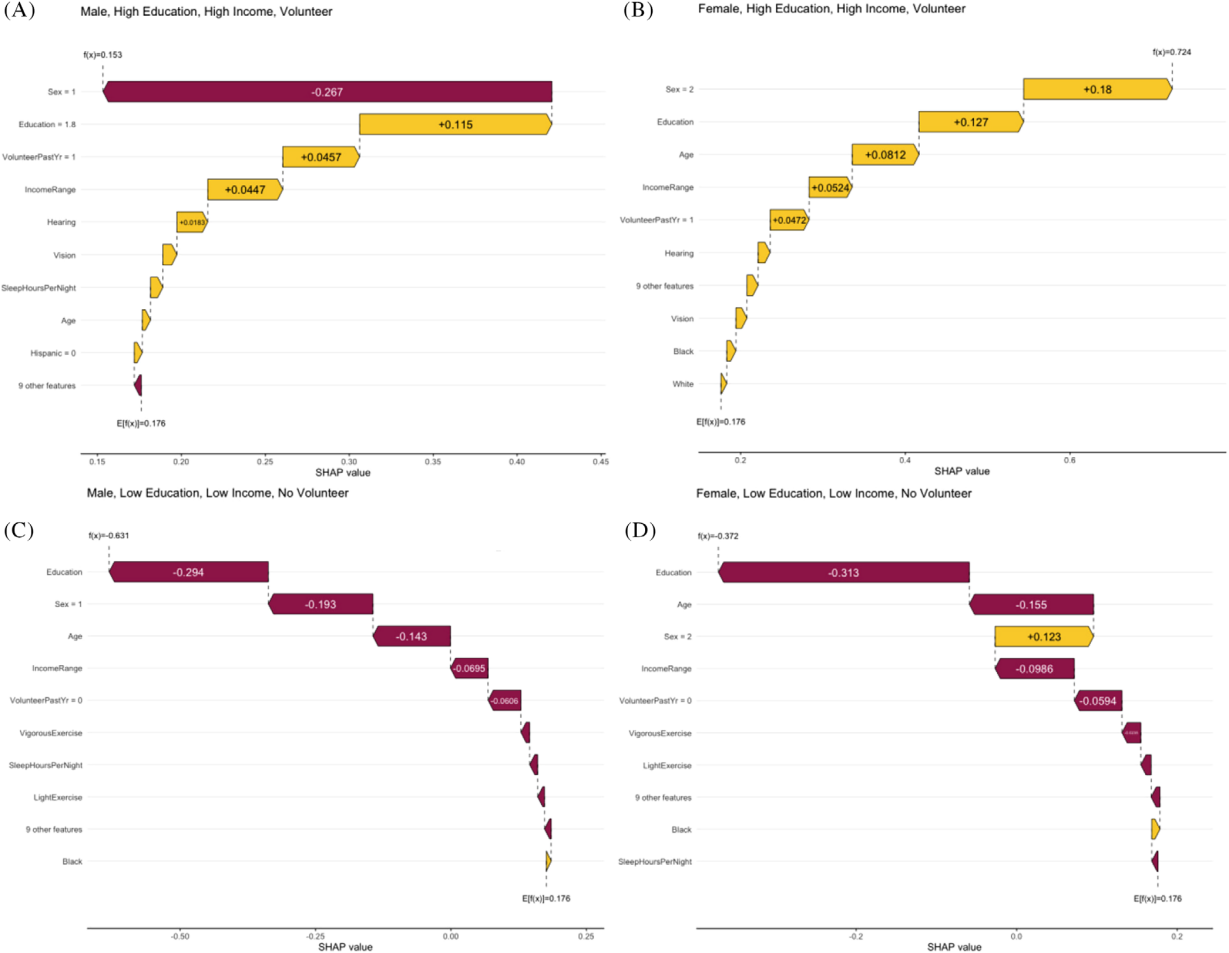
Waterfall plots showing group impacts of LEFs. Waterfall plots such as these can be powerful tools in determining individual LEFs to target in personalized ADRD risk reduction interventions. The top figures show males (A, *n* = 31) and females (B, *n* = 31) with the highest quartiles for education and income engaged in volunteering. The bottom figures show males (C, *n* = 21) and females (D, *n* = 50) with the lowest quartile for education and income and no volunteering. Overall memory score prediction is given by f(*x*) in each graph. E[f(*x*)] = 0.176 is the mean predicted memory score across the whole data before adjustment by impacts for individual variables. Individual signed contributions (SHAP values) are printed inside the impact arrows. Vertical ranking is by magnitude of mean signed SHAP values. ADRD, Alzheimer's disease and related dementias; LEF, life exposure factor; SHAP, Shapley Additive exPlanation.

Figure 4 presents sample waterfall plots to illustrate the participant‐specific (local) explanatory power of our model. These figures illustrate the model's capacity for local importance rankings and individualized predictions, showcasing the interpretability and granular detail in our findings. The subgroups are selected to highlight groups of the lowest and highest quartiles of education and income for males and females. In the top row (Figure 4A,B), males and females with similarly high education levels (post college), and similar income, and engaged in volunteering activities show opposite impacts of sex (negative for males, positive for females) in the context of otherwise positive LEF impacts for most other features. The second set of figures (Figure 4C,D) shows males and females with the lowest quartile of education and income and no volunteering. In these groups, sex still influences memory, with roughly the same magnitudes as the corresponding values in the high‐education pair, but its relative importance decreases for both groups, overshadowed by education in the males and education and age for the females.

## 4 DISCUSSION

Using regression tree machine learning analyses, we examined the relative importance of demographic, socioeconomic, and various well‐known modifiable dementia risk factors in predicting memory performance in a large, diverse cohort of older adults. As expected, age and sex emerged as the strongest predictors, followed by education, volunteering, and income. These findings reinforce previous research^1^, ^2^ highlighting the critical role of demographic and socioeconomic factors in cognitive aging, particularly when considering differences by sex and ethnicity. Our study adds to the literature by systematically ranking these factors based on their global absolute “importance” while also showing their local signed “impact” on memory performance, a sensitive marker of early cognitive decline,^41^ rather than their influence on dementia prevalence.

### 4.1 Demographic and socioeconomic contributors to memory

Age had the highest or second highest importance for memory across all analyses, reinforcing its well‐documented association with cognitive decline.^42^ Interestingly, among males, age was second in importance to sex, while the reverse was true for females. Sex differences were also evident, with females showing a marked advantage in memory. This finding aligns with prior research^43^ suggesting that women tend to outperform men in memory tasks.^24^, ^44^

Among the socioeconomic variables, education emerged as a significant impact on memory performance, particularly for females and Hispanic participants, where its global importance was greater than for other ethnic groups. This aligns with previous research highlighting education's prominent role in reducing ADRD risk in these populations.^6^, ^45^ Education is closely linked with memory performance,^46^ with its effects potentially occurring through both indirect and direct pathways. Indirectly, education is associated with brain measures that support cognition,^47^ while directly, it fosters cognitive skills in early adulthood that support memory in later life.^46^ Additionally, education may enhance cognitive reserve, the ability to sustain cognition in the face of brain pathology,^48^, ^49^ though this relationship is complex and nuanced.^50^, ^51^

Income was next in importance to education for memory performance, with a greater importance observed in females than males. This suggests that financial stability may play a more significant role in mitigating cognitive decline among women. A recent review highlighted the effects of childhood poverty on children's neural development and cognition, emphasizing the importance of early‐life interventions.^52^ While many studies have examined income and education as indicators of adult socioeconomic status (SES), SES has been consistently linked to better cognitive function in later life^53^, ^54^, ^55^, ^56^ and improved prospective memory performance.^57^ Recent research found that individuals with higher SES experience slower biological aging, though the association of biological aging and memory trajectories varies across ethnic and sex categories.^58^ These findings provide a compelling explanatory framework for the strong influence of income in our models.

### 4.2 Lifestyle and health‐related factors

LEFs such as volunteering, exercise, and sleep consistently ranked among the most important predictors of memory performance. Notably, volunteering has not been widely examined in previous studies of ADRD risk,^1^, ^2^ highlighting the need for future research to determine whether interventions targeting these factors could improve memory performance and reduce ADRD risk.^59^, ^60^

Volunteering may enhance cognitive reserve through increased physical activity, social interactions, and mental stimulation.^61^ Our results reinforce previous findings about the importance of volunteering to cognitive functioning^62^ and dementia risk,^59^ as it consistently emerged as one of the top factors influencing memory, suggesting that it may subserve biological effects that promote brain health.^62^ The slightly greater impact observed in females aligns with previous findings,^61^ but the relatively constant impact of volunteering across ethnic groups may suggest a difference from existing literature.^63^

Recent meta‐analyses and literature reviews suggest that exercise is associated with positive cognitive benefits in older adults.^64^, ^65^, ^66^, ^67^ In our models, vigorous exercise demonstrated a moderate but consistent positive impact on memory, supporting existing evidence and expanding it to suggest that a moderate frequency of vigorous exercise may be more beneficial than either excessive or insufficient vigorous exercise. Similarly, moderate sleep duration was more beneficial than excessive or insufficient sleep duration. Studies suggest that adequate sleep duration (at least 7 hours) and high‐quality sleep (e.g., greater sleep efficiency) are associated with reduced risk of ADRD.^68^ Additionally, the connection between sleep and memory is well documented,^69^ as sleep plays a crucial role in supporting neural mechanisms involved in memory encoding, consolidation, and retrieval.^69^

Sensory health factors, including hearing and vision, also had a meaningful impact on memory performance, with hearing being more influential for males and vision for females. This discrepancy may reflect sex‐based differences in sensory processing and compensatory strategies for cognitive tasks. In contrast, factors like smoking, alcohol use, and TBI had lesser importance for memory, despite their well‐documented effect on many health outcomes.^70^ While these variables were less influential in our study compared to education, income, volunteering, and sleep, their associations with memory performance remained consistent with the extant literature.^59^, ^60^ Both smoking and excessive alcohol use had modest but clearly negative impacts on memory. The difference in their global impact rankings compared to previous studies of ADRD risk^1^, ^2^ may stem from the difference between our outcomes (memory vs. ADRD risk).

### 4.3 Ethnic variations in memory predictors

In the full and sex‐stratified models, ethnicity had modest global importance, with generally positive local impact on memory for Asian and White participants and generally negative for Hispanic and Black participants. Notably, education was particularly influential (Figure 3) for Hispanic participants, consistent with findings from Nianogo et al.^2^ Ethnic identity serves as a proxy for a range of sociocultural factors, including structural racism and discrimination,^71^ that underlie disparities in cognition and brain health.^72^ The overall impact of ethnic group membership on memory performance may indicate the net effects of a combination of factors, such as experiences of discrimination,^73^ lower SES and associated stress,^72^ adverse childhood experiences,^28^ occupational complexity,^74^ and cardiometabolic health,^75^ that impact cognitive outcomes. Our findings suggest that future research could leverage similar methods to explore how socioeconomic and cultural factors (e.g., childhood SES, structural discrimination) contribute to cognitive disparities. Importantly, despite the modest impacts of ethnic identity markers in our models, our results consistently showed that education, income, volunteering, hearing, vision, and sleep were among the most important modifiable factors across groups. These findings underscore the critical role of socioeconomic and lifestyle factors in supporting cognitive health across diverse populations.

### 4.4 Implications, strengths, limitations, and future directions

This study has important implications for public health and intervention strategies aimed at mitigating cognitive decline. Given the strong influence of education, policies that promote access to quality education across the lifespan could enhance cognitive resilience. Similarly, initiatives encouraging social engagement through volunteering and physical activity, along with sleep interventions, may serve as protective factors for memory performance. Addressing sensory health, particularly through early intervention for hearing and vision impairments, may also contribute to memory performance in aging populations.

Our study has considerable strengths. These include the powerful XGBoost technique combined with SHAP value explanations and a large and ethnically diverse cohort with a rich set of dementia risk factors harmonized across two datasets. We ranked the global importance of risk factors for memory, as is done in ordinary regression models, while also accounting for all possible interactions (leading to the local explanations of the SHAP values), which is difficult or impossible to achieve in regression. While our global findings are broadly consistent with extant literature, we also generated individual explanations (i.e., bee swarm and waterfall plots) that support the findings’ potential clinical utility in personalized interventions tailored to healthy cognitive aging and ADRD risk reduction. Our study has limitations related to the self‐reported nature of the risk factors, as well as the potential for some of the factors (i.e., exercise, smoking, alcohol consumption, and TBI) to rank lower on global importance as a function of the measurement tools used to assess them. Almost half of our sample was Black adults, while other ethnic groups were less represented, potentially impacting our findings. Similarly, the data come from Kaiser Permanente in California, limiting the generalizability of our data. Moreover, we chose only 12 risk factors and did not examine variables such as cardiovascular disease or other health indicators known to affect memory performance that were unavailable or had too many missing data points. Future research should explore potential causal mechanisms underlying these associations, incorporating longitudinal designs to assess changes over time, including more biological variables and ADRD biomarkers. Additionally, investigating interactions among demographic, lifestyle, and health factors using causal inference techniques could further elucidate pathways to cognitive resilience. Expanding analyses to include additional social determinants, such as neighborhood environments and health‐care access, may provide a more comprehensive understanding of factors influencing memory outcomes.

## 5 CONCLUSION

This study highlights the dominant role of demographic and socioeconomic factors in memory prediction, with notable contributions from lifestyle and health‐related variables. The findings emphasize the need for targeted interventions that consider sex and ethnic disparities, as well as broader socioeconomic and socioenvironmental determinants of cognitive health. By leveraging machine learning models such as XGBoost, future research can enhance the precision of cognitive risk assessments and develop more personalized strategies to promote healthy cognitive aging.

## CONFLICT OF INTEREST STATEMENT

The authors have no competing interests to declare. Author disclosures are available in the supporting information.

## CONSENT STATEMENT

All data was pre‐acquired in existing studies, and no consent statements are required.

## Supporting information



Supporting Information

Supporting Information
