# Supplementary material for: Modeling the importance of life exposure factors on memory performance in diverse older adults: A machine learning approach
Source: Alzheimers Dement. 2025 Aug 20;21(8):e70428. doi: 10.1002/alz.70428 (PMC12365611; doi:10.1002/alz.70428)
Supplement: Supplementary file 1 — Supporting Information [file ALZ-21-e70428-s002.docx]

**Table S1. Missing Variable Analysis**

| Variable | Missing (n) | Missing Percent |
| --- | --- | --- |
| Alcohol Exceed Threshold | 601 | 26.7 |
| Income Range | 252 | 11.2 |
| Traumatic Brain Injury | 63 | 2.8 |
| Sleep Quality | 49 | 2.2 |
| Vigorous Exercise | 45 | 2.0 |
| Light Exercise | 37 | 1.6 |
| Hearing | 34 | 1.5 |
| Vision | 34 | 1.5 |
| Sleep Hours Per Night | 29 | 1.3 |
| Ever Smoked | 24 | 1.1 |
| Education Years | 11 | 0.5 |
| Volunteering in Past Year | 4 | 0.2 |
| Memory | 0 | 0 |
| Age | 0 | 0 |
| Sex | 0 | 0 |
| Asian | 0 | 0 |
| African American | 0 | 0 |
| Hispanic | 0 | 0 |
| White | 0 | 0 |

Note: Percentages calculated based on total sample size (N = 2,245). Variables are ordered by frequency of missing data in descending order.

**Table S2a. Tukey HSD Analysis Results for Sex Differences**

| Variable | Mean Difference | SE | q-value | MSW | Male Mean | Female Mean | Sig |
| --- | --- | --- | --- | --- | --- | --- | --- |
| Memory | -0.497 | 0.037 | 13.45 | 0.719 | -0.140 | 0.357 | *** |
| Income Range | 0.499 | 0.045 | 11.12 | 0.942 | 0.312 | -0.188 | *** |
| Vigorous Exercise | 0.291 | 0.044 | 6.67 | 0.981 | 0.182 | -0.109 | *** |
| Hearing | -0.230 | 0.044 | 5.27 | 0.988 | -0.144 | 0.086 | *** |
| Sleep Quality | -0.220 | 0.044 | 5.02 | 0.989 | -0.138 | 0.082 | *** |
| Smoke Ever | 0.108 | 0.022 | 4.98 | 0.245 | 0.517 | 0.409 | *** |
| Traumatic Brain Injury | 0.082 | 0.018 | 4.54 | 0.168 | 0.268 | 0.186 | *** |
| Volunteer Past Year | -0.082 | 0.022 | 3.79 | 0.248 | 0.429 | 0.511 | ** |
| Education | 0.155 | 0.044 | 3.56 | 0.995 | 0.097 | -0.058 | ** |
| Age | 0.147 | 0.044 | 3.37 | 0.995 | 0.092 | -0.055 | ** |
| Alcohol Exceed Threshold | 0.033 | 0.015 | 2.13 | 0.088 | 0.118 | 0.086 | * |
| Sleep Hours Per Night | 0.074 | 0.044 | 1.68 | 0.999 | 0.046 | -0.028 | ns |
| Light Exercise | 0.038 | 0.044 | 0.87 | 1.000 | 0.024 | -0.014 | ns |
| Vision | 0.020 | 0.044 | 0.44 | 1.000 | 0.012 | -0.007 | ns |

**Note: Glossary**

MSW= Mean Square Within (measure of within-group variance); Sig = significance.

SE = Standard Error

Sig = Significance

**Interpretation**:

For two groups (male vs female), the Tukey HSD q-value critical points are:

- - q > 4.40: p < 0.001 (***)
  - q > 3.64: p < 0.01 (**)
  - q > 2.77: p < 0.05 (*)
- Mean Differences:
  - Positive values (blue): Males score higher
  - Negative values (brown): Females score higher
- All variables are standardized (z-scores)

**Key Findings**:

1. Very Large Differences (q > 10):
   - Memory (q = 13.45): Strong female advantage
   - Income Range (q = 11.12): Strong male advantage
2. Large Differences (5 < q < 10):
   - Vigorous Exercise (q = 6.67): Male advantage
   - Hearing (q = 5.27): Female advantage
   - Sleep Quality (q = 5.02): Female advantage
3. Moderate Differences (2.77 < q < 5):
   - Smoking, TBI, Volunteering, Education, Age
4. Non-significant Differences (q < 2.77):
   - Sleep Hours, Light Exercise, Vision

**Table S2b. Tukey HSD Analysis Results for Ethnic Differences**

| Variable | Comparison | Difference | SE | q-value | Sig |
| --- | --- | --- | --- | --- | --- |
| Large Differences (q > 9.0) | | | | | |
| Age | AA-White | -0.703 | 0.052 | 13.43 | *** |
| Sleep Hours | AA-White | -0.645 | 0.054 | 12.05 | *** |
| Education | Asian-Hispanic | 0.817 | 0.070 | 11.59 | *** |
| Age | AA-Hispanic | -0.597 | 0.059 | 10.09 | *** |
| Education | Hispanic-White | -0.687 | 0.068 | 10.14 | *** |
| Age | Asian-AA | 0.546 | 0.056 | 9.81 | *** |
| **Moderate Differences (6.0 < q < 9.0)** | | | | | |
| Alcohol Exceed | AA-White | -0.170 | 0.019 | 8.76 | *** |
| Income Range | Asian-Hispanic | 0.576 | 0.077 | 7.50 | *** |
| Education | Asian-AA | 0.396 | 0.057 | 6.98 | *** |
| Education | AA-Hispanic | 0.421 | 0.060 | 6.97 | *** |
| Sleep Hours | Asian-White | -0.446 | 0.065 | 6.86 | *** |
| Alcohol Exceed | Asian-White | -0.149 | 0.023 | 6.61 | *** |
| Smoke Ever | Asian-White | -0.203 | 0.033 | 6.18 | *** |
| **Small but Significant Differences (4.37 < q < 6.0)** | | | | | |
| Income Range | Asian-AA | 0.355 | 0.061 | 5.78 | *** |
| Sleep Hours | AA-Hispanic | -0.336 | 0.061 | 5.55 | *** |
| Hearing | AA-White | 0.285 | 0.055 | 5.18 | *** |
| Income Range | Hispanic-White | -0.378 | 0.074 | 5.14 | *** |
| Education | AA-White | -0.266 | 0.053 | 5.00 | *** |
| Smoke Ever | Asian-Hispanic | -0.167 | 0.036 | 4.61 | *** |
| TBI | AA-Hispanic | -0.122 | 0.026 | 4.72 | *** |
| Sleep Hours | Hispanic-White | -0.309 | 0.068 | 4.54 | *** |
| Sleep Quality | Asian-AA | -0.269 | 0.059 | 4.55 | *** |
| Memory | Asian-Hispanic | 0.289 | 0.064 | 4.53 | *** |
| Volunteer | AA-Hispanic | 0.138 | 0.031 | 4.45 | *** |
| Smoke Ever | Asian-AA | -0.129 | 0.029 | 4.46 | *** |
| Alcohol Exceed | Hispanic-White | -0.109 | 0.025 | 4.37 | *** |

**Note: Glossary**

AA = African American

SE = Standard Error

Sig = Significance

TBI = Traumatic Brain Injury

Key Findings:

1. Demographic Variables

- Age shows the largest disparities:
  - AA participants significantly younger than White (q = 13.43), Hispanic (q = 10.09), and Asian (q = 9.81) participants
  - No significant differences among Asian, Hispanic, and White participants
- Education shows substantial differences:
  - Asian participants have significantly higher education than Hispanic (q = 11.59) and AA (q = 6.98) participants
  - White participants have significantly higher education than Hispanic (q = 10.14) and AA (q = 5.00) participants

2. Health Behaviors

- Sleep Hours shows consistent patterns:
  - White participants report significantly more sleep than AA (q = 12.05), Asian (q = 6.86), and Hispanic (q = 4.54) participants
- Alcohol and Smoking:
  - White participants report higher alcohol consumption than AA (q = 8.76) and Asian (q = 6.61) participants
  - Asian participants report lower smoking rates than all other groups

3. Socioeconomic Factors

- Income Range shows consistent disparities:
  - Asian participants report higher income than Hispanic (q = 7.50) and AA (q = 5.78) participants

**Table S2c. ANOVA Results for Sex Differences**

| Variable | F value | df | Male Mean | Female Mean | Sig |
| --- | --- | --- | --- | --- | --- |
| Memory | 180.85 | 1, 2243 | -0.140 | 0.357 | *** |
| Income Range | 123.70 | 1, 1992 | 0.312 | -0.188 | *** |
| Vigorous Exercise | 44.45 | 1, 2198 | 0.182 | -0.109 | *** |
| Hearing | 27.73 | 1, 2209 | -0.144 | 0.086 | *** |
| Sleep Quality | 25.25 | 1, 2194 | -0.138 | 0.082 | *** |
| Smoke Ever | 24.80 | 1, 2219 | 0.517 | 0.409 | *** |
| TBI | 20.62 | 1, 2180 | 0.268 | 0.186 | *** |
| Volunteer Past Year | 14.33 | 1, 2239 | 0.429 | 0.511 | *** |
| Education | 12.70 | 1, 2232 | 0.097 | -0.058 | *** |
| Age | 11.34 | 1, 2243 | 0.092 | -0.055 | *** |
| Alcohol Exceed Thresh | 4.55 | 1, 1642 | 0.118 | 0.086 | * |
| Sleep Hours Per Night | 2.82 | 1, 2214 | 0.046 | -0.028 | ns |
| Light Exercise | 0.76 | 1, 2206 | 0.024 | -0.014 | ns |
| Vision | 0.20 | 1, 2209 | 0.012 | -0.007 | ns |

**Note: Glossary**

Df = degrees of freedom

Sig = Significance

TBI = Traumatic Brain Injury

**Table S2d. ANOVA Results for Ethnic Differences**

| Variable | F value | df | Asian Mean | AA Mean | Hispanic Mean | White Mean | Sig |
| --- | --- | --- | --- | --- | --- | --- | --- |
| Age | 81.13 | 3, 2241 | 0.198 | -0.349 | 0.248 | 0.354 | *** |
| Education | 53.90 | 3, 2230 | 0.330 | -0.067 | -0.487 | 0.200 | *** |
| Sleep Hours Per Night | 50.00 | 3, 2212 | -0.033 | -0.232 | 0.104 | 0.413 | *** |
| Alcohol Exceed Thresh | 26.75 | 3, 1640 | 0.072 | 0.051 | 0.113 | 0.222 | *** |
| Income Range | 21.53 | 3, 1990 | 0.290 | -0.066 | -0.286 | 0.092 | *** |
| Smoke Ever | 13.81 | 3, 2217 | 0.322 | 0.451 | 0.489 | 0.525 | *** |
| TBI | 12.65 | 3, 2178 | 0.177 | 0.174 | 0.295 | 0.278 | *** |
| Hearing | 12.03 | 3, 2207 | -0.099 | 0.141 | -0.077 | -0.143 | *** |
| Sleep Quality | 9.27 | 3, 2192 | -0.150 | 0.119 | -0.114 | -0.030 | *** |
| Light Exercise | 8.82 | 3, 2204 | 0.147 | -0.102 | -0.049 | 0.114 | *** |
| Memory | 8.43 | 3, 2241 | 0.247 | 0.194 | -0.041 | 0.210 | *** |
| Volunteer Past Year | 7.44 | 3, 2237 | 0.453 | 0.516 | 0.378 | 0.505 | *** |
| Vision | 5.19 | 3, 2207 | -0.012 | -0.064 | -0.021 | 0.152 | ** |
| Vigorous Exercise | 2.34 | 3, 2196 | 0.030 | -0.040 | -0.055 | 0.092 | ns |

**Note: Glossary**

AA = African American

SE = Standard Error

Sig = Significance

TBI = Traumatic Brain Injury

Note: * p < 0.05, ** p < 0.01, *** p < 0.001, ns = not significant. All variables are standardized (z-scores).

**Key Findings**

- Sex Differences:
- Largest effects (F > 100):
  - Memory (F = 180.85): Females higher
  - Income Range (F = 123.70): Males higher
- No significant differences in:
  - Vision
  - Light Exercise
  - Sleep Hours Per Night
- 2. Race/Ethnicity Differences:
- Largest effects (F > 50):
  - Age (F = 81.13): White highest, Black lowest
  - Education (F = 53.90): Asian highest, Hispanic lowest
  - Sleep Hours (F = 50.00): White highest, Black lowest
- Only Vigorous Exercise showed no significant racial/ethnic differences

**Table S3. Best Model Hyperparameters**

| Parameter | Value |
| --- | --- |
| mtry | 4 |
| trees | 584 |
| min_n | 66 |
| tree_depth | 6 |
| learn_rate | 0.0082 |
| loss_reduction | 0.0020 |
| sample_size | 0.71 |

**Note 1: Glossary**

AA= African American

learn_rate – controls how much each tree contributes to ensemble prediction. Also known as “eta”

loss_reduction – minimum loss reduction required for making a split. Also known as “gamma”

min_n – minimum number of observations needed at a node before further splitting can occur

mtry – number of variables randomly sampled as candidates at each split in the trees

sample_size – proportion of the data used to train each tree

trees – number of trees in the ensemble of the model

tree_depth – maximum depth allowed for each tree

**Note 2:** Values represent the optimal hyperparameters found during model tuning. Learn_rate, loss_reduction, and sample_size are rounded to 4 decimal places for presentation.
